# Quantitative Genetic Modeling of the Parental Care Hypothesis for the Evolution of Endothermy

**DOI:** 10.3389/fphys.2017.01005

**Published:** 2017-12-11

**Authors:** Leonardo D. Bacigalupe, Allen J. Moore, Roberto F. Nespolo, Enrico L. Rezende, Francisco Bozinovic

**Affiliations:** ^1^Instituto de Ciencias Ambientales y Evolutivas, Facultad de Ciencias, Universidad Austral de Chile, Valdivia, Chile; ^2^Department of Genetics, University of Georgia, Athens, GA, United States; ^3^Departamento de Ecología, Facultad de Ciencias Biológicas, Center of Applied Ecology and Sustainability, Pontificia Universidad Católica de Chile, Santiago, Chile; ^4^Facultad de Ecología y Recursos Naturales, Universidad Andres Bello, Santiago, Chile

**Keywords:** endothermy, parental care, quantitative genetics, daily energy expenditure, maternal effects

## Abstract

There are two heuristic explanations proposed for the evolution of endothermy in vertebrates: a correlated response to selection for stable body temperatures, or as a correlated response to increased activity. Parental care has been suggested as a major driving force in this context given its impact on the parents' activity levels and energy budgets, and in the offspring's growth rates due to food provisioning and controlled incubation temperature. This results in a complex scenario involving multiple traits and transgenerational fitness benefits that can be hard to disentangle, quantify and ultimately test. Here we demonstrate how standard quantitative genetic models of maternal effects can be applied to study the evolution of endothermy, focusing on the interplay between daily energy expenditure (DEE) of the mother and growth rates of the offspring. Our model shows that maternal effects can dramatically exacerbate evolutionary responses to selection in comparison to regular univariate models (breeder's equation). This effect would emerge from indirect selection mediated by maternal effects concomitantly with a positive genetic covariance between DEE and growth rates. The multivariate nature of selection, which could favor a higher DEE, higher growth rates or both, might partly explain how high turnover rates were continuously favored in a self-reinforcing process. Overall, our quantitative genetic analysis provides support for the parental care hypothesis for the evolution of endothermy. We contend that much has to be gained from quantifying maternal and developmental effects on metabolic and thermoregulatory variation during adulthood.

## Introduction

The evolution of endothermy in birds and mammals is one of the most puzzling topics in evolutionary physiology (Ruben, 1995; see reviews in Hayes and Garland, 1995; Koteja, 2004; Kemp, 2006; Nespolo et al., 2011). Although endothermy has evolved in many taxonomic groups from plants to insects, birds and mammals are unique because they are able to maintain elevated body temperatures at rest employing the heat produced mainly in the visceral organs (heart, kidneys, liver, intestines) instead of muscle contraction (Ruben, 1995). Those organs have high metabolism per unit of tissue and thus contribute disproportionately to the maintenance metabolism, or basal metabolic rate (BMR), which ultimately determines endothermy (Konarzewski and Diamond, 1995). Despite its multiple benefits, however, organisms are not able to switch-off these expensive organs when they do not need them (e.g., elevated temperatures), and consequently their constant maintenance seems a wasteful strategy from an energetic point of view (Koteja, 2004). Given the elevated energy costs associated with endothermy, the selective pressures that have favored the emergence of high-energy turnover rates remain highly controversial (Nespolo et al., 2011).

Several hypotheses have been advanced to explain the evolution of elevated BMR in mammals and birds. Most of the debate revolves around two main hypotheses, namely that endothermy evolved as a correlated response to selection for higher and stable body temperatures on the one hand, or for higher levels of aerobic metabolism for sustained activity on the other hand (Hayes and Garland, 1995; Ruben, 1995; Koteja, 2004; Kemp, 2006). Importantly, a shared characteristic of both hypotheses is that they focus solely on selection of adult organisms and subsequent evolutionary responses. Alternatively, parental care was recently proposed as a major target of selection during the evolution of endothermy (Farmer, 2000; Koteja, 2000, 2004; Clavijo-Baquet et al., 2016), which is not mutually exclusive with the previous scenarios but highlights potential fitness benefits to the offspring that have been previously ignored. Specifically, the selective advantages of decreased mortality of the offspring by means of a faster growth rate, either due to the ability of the parents to control incubation temperature (Farmer, 2000) and/or to provide food (Farmer, 2000; Koteja, 2000), could potentially offset the energy costs associated with a highly active endothermic lifestyle. Therefore, even though different hypotheses do not agree on the proximate mechanism by which a higher metabolic levels evolved, they do agree that to understand the evolution of endothermy we should look at the complete life history of organisms (Koteja, 2000; Clavijo-Baquet and Bozinovic, 2012).

Our aim here is to present a theoretical framework for testing the parental care hypothesis (Farmer, 2000; Koteja, 2000) based on adapting a simple quantitative genetic model of maternal effects develop by Cheverud (1984) and Cheverud and Moore (1994). We work with the premise that parental care is energetically costly fundamentally because providing food to the offspring requires high and sustained locomotor activity fueled by assimilated food (Koteja, 2000, 2004). High energy turnover rates involve processing greater amounts of food and therefore require high capacities in the visceral organs associated with these processes, namely the organs that contribute disproportionately to BMR (Bacigalupe and Bozinovic, 2002; Bacigalupe et al., 2004) and, eventually, to elevated body temperature as heat dissipation decreases with better thermal insulation. This suggests that both the energy costs and the putative benefits of parental care, which involve primarily food provisioning and the ability to control incubation temperatures, can be captured by daily energy expenditure (DEE) of the parents. Therefore, following the approach that is outlined in detail by Cheverud and Moore (1994), we model the phenotypic evolution of offspring growth rate as a partial consequence of mother parental care, expressed as DEE (Koteja, 2000).

This approach follows the theoretical modeling of maternal effects (Kirkpatrick and Lande, 1989; Cheverud and Moore, 1994) or more generally interacting phenotypes (Moore et al., 1997), treating the offspring and maternal traits as different and separate. Thus, traits expressed by the mother provide an environmental influence on offspring but the maternal trait itself can vary due to genetic variation amongst mothers, and is heritable. Thus, there are two important aspects of maternal interacting phenotypes. First, a trait in one individual influences the phenotype of the other. Here, we assume that maternal DEE affects the expression of the offspring trait, but the offspring trait does not influence the maternal DEE. This results in genetic contributions to offspring traits such as growth rate that reflect both direct genetic effects expressed in the offspring and indirect genetic effects arising from DEE expressed in the mother that is acting as an environment on the offspring. Second, selection acts on *both* traits, so that genetic variation in one trait can influence the evolution in a second trait. This can enhance or retard evolution, and the focal trait can evolve even if there are no direct genetic effects for that trait (Moore et al., 1997; Bijma, 2014). The effect discussed here is that the evolution of the offspring trait is influenced by the evolution of maternal DEE as well. Thus, our model is not simply a mathematical treatment of the parental care hypothesis but goes one step further. That is, apart from understanding the consequences of selection acting on parental care only, as proposed by Koteja (2000) and Farmer (2000), we also model the evolutionary consequences of selection acting on offspring growth rates for both offspring and mothers.

For details of the derivations of models of maternal effects and associated selection, the primary literature should be consulted (e.g., Cheverud, 1984; Riska et al., 1985; Kirkpatrick and Lande, 1989; Cheverud and Moore, 1994 and references therein). As detailed in Cheverud (1984) and Cheverud and Moore (1994), the model presented here has the usual assumptions of quantitative genetics (Lynch and Walsh, 1998), including the phenotype is affected by many genes of small additive effect, there is no dominance or epistasis, and selection is weak. We also assume maternal effects are unidirectional (i.e., there are no maternal effects on offspring DEE and there is no feedback from the offspring growth rate to the maternal DEE; see Moore et al., 1997; McGlothlin and Brodie, 2009; Bijma, 2014 for more detailed discussions of other assumptions). These assumptions can be relaxed; particularly the feedback assumption (Riska et al., 1985), but the main message and parameters to measure remain unchanged.

## Maternal effects and endothermy: the model

Here we present a variance component model for the evolution of offspring performance affected by maternal endothermy. This model incorporates social effects; that is, the influence of traits expressed in others that also influence the traits of a focal individual. Such traits, or interacting phenotypes (Moore et al., 1997), are likely common (Bijma, 2014) and have consequences for both selection and inheritance, due to “indirect genetic effects” (Moore et al., 1997; Bijma, 2014). Because there are often prolonged associations between mothers and offspring, as well as prenatal provisioning of the egg by the mother, the most common form of indirect genetic effect are maternal genetic effects arising from maternal effects. It is this form of interacting phenotype model and indirect genetic effect that we consider here. Although we have focused on care by a single sex and interchangeably describe maternal or parental effects, this model can easily be extended to include effects from the father as well as the mother (Cheverud and Moore, 1994).

Because offspring performance and endothermy are both influenced by many different traits within an individual, here we employ the more general variance component model. We believe that this model is heuristically the most easily understood. Importantly, the parameters that our model suggests should be measured are the same regardless of the specific maternal effect model used (McGlothlin and Brodie, 2009). For example, Kirkpatrick and Lande (1989) developed a general model that considers maternal effects and specific traits, and their multivariate model is most appropriate when all traits are known and measured, which is not the case here (see McGlothlin and Brodie, 2009; Bijma, 2014 for a discussion of the difference). Aside from being somewhat more tractable empirically, without knowledge of all the maternal traits that contribute to the offspring phenotype, the most reliable empirical approach is to estimate offspring performance as a way of estimating maternal effects.

### Inheritance when phenotypes interact–indirect genetic effects

Consider a trait that contributes to performance and survival, such as body weight or growth rate. In quantitative genetic terms, such trait can be described in terms of the additive genetic and environmental components that make up that trait:

(1) $$\begin{array}{r} z=a+e \end{array}$$

Where *z* is any phenotype (measured trait), *a* is the additive genetic effect, and *e* is the environmental contribution, or all non-additive genetic effects plus all environmental effects.

Although this is the standard linear model for any trait (Lynch and Walsh, 1998), many focal phenotypes of an individual are influenced by the phenotypes of other individuals in the environment, especially relatives such as mothers. Body weight and growth rate are offspring performance traits, which are often influenced by both the genes expressed in the individual and the environment provided by the mother (or any parental effect). We therefore add subscripts to indicate which trait, maternal or offspring, is being considered where traits expressed in the offspring are given the subscript O and traits expressed in the mother are given the subscript DEE as we are assuming here that DEE is the trait in the mother that influences offspring performance. For example, *z*_*DEE*_ in our model is the maternal DEE influenced by additive genetic effects and non-additive and environmental effects:

(2a) $$\begin{array}{r} z_{DEE}=a_{DEE}+e_{DEE} \end{array}$$

Equation 2a is a standard quantitative genetic description for any phenotypic trait. However, here we are considering offspring traits such as growth rate that may be influenced by the parent in contributions that go beyond genetics; i.e., maternal effects arising from parental care (here, maternal DEE). Thus, the description for the offspring growth rate (*z*_*O*_) includes the contribution of the mother's phenotype:

(2b) $$\begin{array}{r} z_{O}=a_{O}+e_{O}+z_{DEE\left(t-1\right)} \end{array}$$

The new term that is added is *z*_*DEE*_, which reflects that the offspring trait is influenced by additive genetic effects, non-additive and environmental effects, and—subdividing the environmental effect into another specific component—the maternal environment created by DEE of the mother.

(2c) $$\begin{array}{r} z_{O}=a_{O}+e_{O}+a_{DEE\left(t-1\right)}+e_{DEE\left(t-1\right)} \end{array}$$

In Equation 2c, we have further divided the environment provided by the mother [maternal DEE, *z*_*DEE*(*t*−1)_] which itself reflects genetic influences on the mother as well as the environment influenced by the mother. The *t*−1 subscript indicates that these effects were expressed in the previous generation of the mother but are having an influence on the current offspring generation.

**Figure 1:**
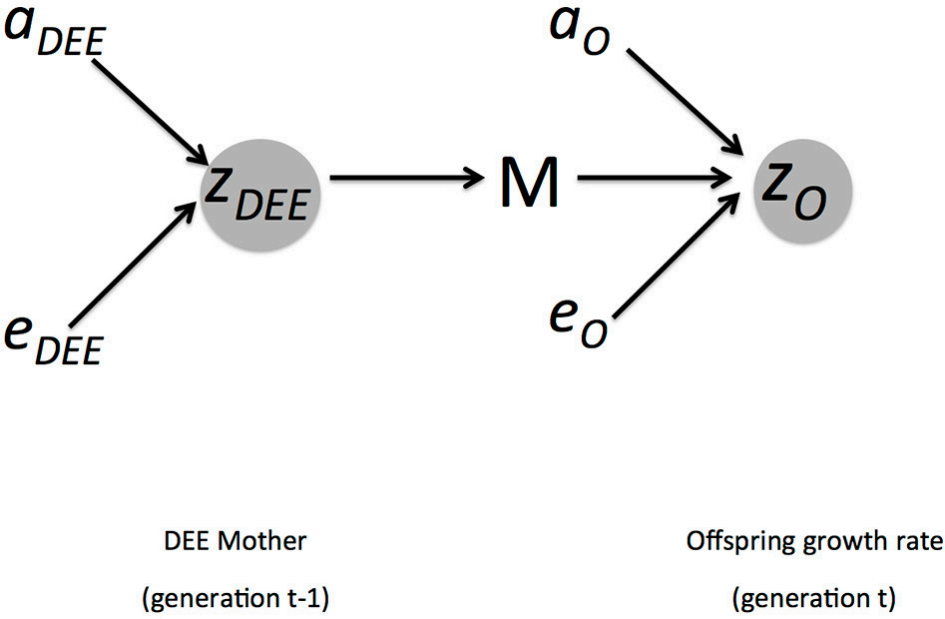
Summary of genetic and environmental influences of mothers DEE (*z*_*DEE*_) on offspring growth rate (*z*_*O*_). Arrows indicate direction of causality. The offspring phenotype is influenced by direct additive genetic effects (*a*_*O*_), non-additive and environmental effects (*e*_*O*_), and the maternal environment M. This is part of the environment of the offspring, but as a maternal trait it is also influenced by direct additive genetic effects (*a*_*DEE*_) and non-additive and environmental ones (*e*_*DEE*_). The covariance (Cov(*A*_*O*_, *A*_*DEE*_), not shown here) between growth rate and DEE arises because the offspring also inherits the DEE genes from the mother.

Equation 2c is a standard and general quantitative genetic model of maternal effects. This phenotypic model can then be used further to consider how traits evolve; i.e., respond to selection and change across generations. For this it is useful to define the total breeding value, *A*, which is calculated from the sum of the individual trait breeding values reflecting the average effect of an individual on the population. In the case of a trait that is influenced by maternal effects:

(3) $$\begin{array}{r} A=A_{O}+{\frac{1}{2}A}_{DEE} \end{array}$$

where ½ reflects the fact that in diploid organisms only ½ of the genes are contributed by each parent. Because we are modeling a maternal trait, we have to account for the fact that only ½ of the genetic influence arises from the mother. We illustrate these influences on phenotypes and inheritance in Figure 1.

### Evolution of interacting phenotypes–direct and correlated responses

Evolutionary change in a trait (symbolized $\Delta \bar{z}$ for change over a single generation) is reflected by two components, inheritance and selection. Using the terminology and equations provided by Lande and Arnold (1983):

(4a) $$\begin{array}{r} \Delta \bar{z}=V_{A}\beta =h^{2}S \end{array}$$

where $\Delta \bar{z}$ is the mean change in a single given phenotype from one generation to the next, *V*_*A*_ is the additive genetic variance (i.e., variance in breeding values, A; Lynch and Walsh, 1998), and β is the selection gradient. This is equivalent to the familiar “breeder's equation,” where the mean change reflects the heritability (*h*^2^) and the selection differential, *S*. Selection gradients and differentials are defined by Lande and Arnold (1983) and a practical description on their measurement and applications is provided in Brodie et al. (1995). Quantitative genetic modeling is facilitated by focusing on selection gradients, which measure the strength of the association between any phenotypic trait and fitness (i.e., the covariance between a trait and fitness) independent of other traits. Alternatively, in terms of evolution of breeding values, this can be expressed as a change in the total breeding value after selection.

(4b) $$\begin{array}{r} \Delta \bar{z}=\Delta \bar{A} \end{array}$$

We can thus substitute Equations 2a–c for *z*, or Equation 3 for *A*, to calculate potential evolution.

The Lande and Arnold (1983) equation is easily generalizable to multiple genetic influences; i.e., we can generalize the genetic contributions beyond simple direct genetic effects and additive genetic variance. Any given trait may share genetic influences with other traits through pleiotropy or linkage disequilibrium, and therefore the evolution of that trait is influenced by changes in genetically correlated traits. Under these conditions, we should consider all the combined genetic contributions; i.e., both genetic variances and covariances. Thus, we must consider genetics of multiple traits even when examining the evolution of a single trait. Furthermore, the traits should be measured on a common scale, typically where the mean = 0 and variance = 1, after first being transformed (when necessary) to meet the assumption of normality. Such standardization of phenotypic traits also results in standardized regression coefficients (Lande and Arnold, 1983), which also allows us to compare evolutionary change in phenotypic standard deviation and facilitates comparisons.

When maternal effects are present, we must consider the effects of genes that influence both the maternal trait and the offspring trait, because any given individual will carry genes for both (Equation 3; Figure 1). We can still consider single trait evolution, but now we can include correlated responses to selection due to genetic covariances arising from linkage disequilibrium or pleiotropy. Thus, considering Equation 2b or 3, we can now consider how maternal traits that influence offspring might change alongside the focal trait of interest in offspring because mothers and offspring share genetic influences.

As a first step, we can consider the evolution of each trait separately, reflecting just direct selection on that trait. Beginning with the offspring trait *z*_*O*_, corresponding to growth rate, its selection gradient β_*O*_ and substituting from the equations for the phenotypes (Equation 2c) we obtain:

(5) $$\begin{array}{r} \Delta {\bar{z}}_{O}=\;\left[V_{AO}+\frac{3}{2}Cov\left(A_{O},A_{DEE}\right)+\frac{1}{2}V_{A\left(DEE\right)}\right]\beta_{O} \end{array}$$

Equation 5 illustrates how direct selection on the offspring growth rate *z*_*O*_ can result in complex evolutionary responses due to maternal effects (Equation 4). The coefficients 3/2 and 1/2 arise from the relatedness of mothers and offspring (*r* = 0.5), thus only ½ of the genes in the offspring were derived from the mother.

This equation reflects several aspects of maternal effects. In particular, genetic variation in the focal individual (here, the offspring) reflects traits that have influences in two generations.

Moreover, this equation describes the change in growth rates of the offspring arising from direct selection on this trait. There are two important outcomes illustrated by this model. First, the evolutionary response of offspring growth rate $\Delta {\bar{z}}_{O}$ does not depend only on the presence of additive genetic effects on this trait, as would be expected under the standard quantitative genetic model that assumes that *h*^2^ = *V*_*AO*_/*V*_*ZO*_ (or *h*^2^ = *V*_*A*_ if the phenotypic variance is scaled to 1). Interestingly, this equation also shows that heritable variation in the maternal trait [i.e., V_*A*(*DEE*)_] can lead to evolutionary change $\Delta {\bar{z}}_{O}=\;\frac{1}{2}V_{A\left(DEE\right)}\beta_{O}$ even in the absence of genetic variation in growth rates! In eco-physiological terms, the effect occurs because growth rates are partly mediated by DEE and the amount of parental investment (Equations 2b,c), hence selection for faster growth rates can elicit an evolutionary response by favoring higher levels of parental investment. Second, selection on offspring β_*O*_ is filtered through the genetic covariance Cov(*A*_*O*_, *A*_*DEE*_), and consequently the response to selection on offspring growth rate will be affected by the sign and magnitude of this correlation. The result of any non-zero covariance is, therefore, that selection on the offspring will produce a correlated response on mother's DEE (i.e., when female offspring become mothers in the future generation *t* + 1) and selection on DEE in the maternal generation will produce an evolutionary response in offspring growth rate.

We next consider how selection would influence DEE in the mothers. We are assuming there is no feedback between offspring and the mother, in such a way that the mother's DEE affects offspring growth rate, but the offspring growth rate does not affect mother's DEE. Although we realize this might not always be the case (i.e., a demanding offspring might induce higher levels of activity on mothers and thus on her DEE) including these reciprocal effects tend to accelerate the rate of evolution even further (Moore et al., 1997). In this context, the evolution of DEE is described by the standard model:

(6) $$\begin{array}{r} \Delta {\bar{z}}_{DEE}=\;V_{A\left(DEE\right)}\beta_{DEE} \end{array}$$

where *V*_*A*(*DEE*)_ is the additive genetic variance of maternal DEE and β_*DEE*_ the selection gradient acting on DEE.

Having analyzed how growth rates and DEE should respond to, respectively, directional selection β_*O*_ in the offspring (generation *t*) and β_*DEE*_ in the parents (generation *t*–1), we can now assess how these traits respond in tandem to both selective pressures. In this case, the total response depends on the selection gradients and both direct and correlated responses, or:

(7a) $$\begin{array}{l} \Delta {\bar{z}}_{O}=\;\left[V_{AO}+\frac{3}{2}Cov\left(A_{O},A_{DEE}\right)+\frac{1}{2}V_{A\left(DEE\right)}\right]\beta_{O}\\ +\left[V_{A\left(DEE\right)}+Cov\left(A_{O},A_{DEE}\right)\right]\frac{1}{2}\beta_{DEE} \end{array}$$

(7b) $$\begin{array}{l} \Delta {\bar{z}}_{DEE}=\;\left[\frac{1}{2}V_{A\left(DEE\right)}+Cov\left(A_{O},A_{DEE}\right)\right]\beta_{O}\\ +\left[V_{A\left(DEE\right)}\right]\frac{1}{2}\beta_{DEE} \end{array}$$

**Figure 2:**
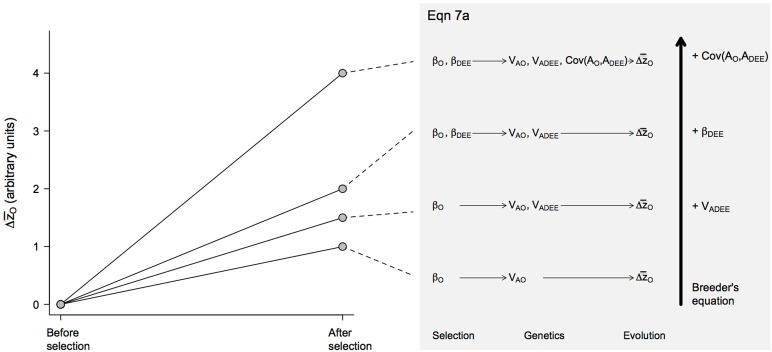
Predicted evolutionary responses of offspring growth rates ($\Delta {\bar{z}}_{O}$) in different evolutionary scenarios. The magnitude of the response was estimated from Equation 7a, with the presence or absence of different effects—*V*_*A*(*DEE*)_, β_*DEE*_ and Cov(*A*_*O*_, *A*_*DEE*_)—included by setting these coefficients to 1 or 0, respectively. Similarly, the coefficients *V*_*AO*_ and β_*O*_ that were always present in the model were set to 1. The resulting evolutionary scenarios are illustrated with the diagrams on the right size, which increase in complexity from bottom to top as an increasing number of effects are incorporated in the model.

**Figure 3:**
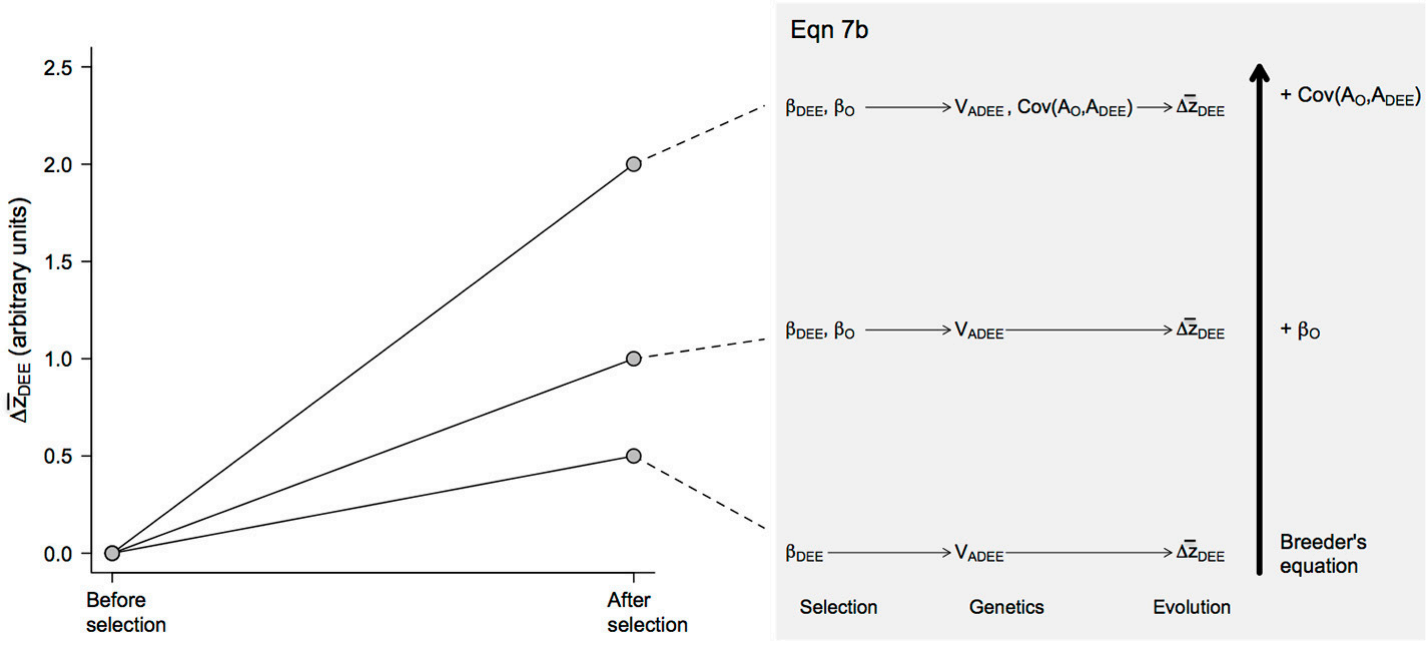
Predicted evolutionary responses of maternal DEE ($\Delta {\bar{z}}_{DEE}$) in different evolutionary scenarios. The magnitude of the response was calculated from Equation 7b following the same procedure detailed for offspring growth rates (Figure 2). Note that the magnitude of the response is smaller than in offspring growth rates because selection is assumed to impact only mothers, not fathers, resulting in some terms being multiplied by ½ as is the case with breeder's equation when β_*O*_ is set to 0 (see Equation 7b).

These equations highlight the importance of maternal effects in the context of the evolution of endothermy, showing that selection on growth rates (β_*O*_) and maternal DEE (β_*DEE*_) should generally result in a correlated response in the other trait for two reasons. First, a correlated response is expected if there is a non-zero genetic covariance Cov(*A*_*O*_, *A*_*DEE*_), which is by no means surprising. Second, correlated responses could occur even in the absence of genetic covariance due to maternal effects *sensu stricto* (i.e., environmental effects). In this scenario, for instance, selection β_*DEE*_ for elevated maternal DEE should elicit an evolutionary response in $\Delta {\bar{z}}_{O}$ (Figure 2) because parental investment is expected to increase at a rate proportional to ½ *V*_*A*(*DEE*)_(Equation 7a). Alternatively, selection for higher offspring growth rates β_*O*_ should affect maternal DEE (Equation 7b) because, as one selects for offspring that grow faster at generation *t*, one is indirectly favoring increased parental investment at generation *t*–1 and genes associated with DEE (Figure 3). However, because selection occurs in different generations, there will be time-lags and evolution may not be direct (see Kirkpatrick and Lande, 1989 for a description of the effects of time-lags, which tend to be short term). Long-term evolution is not affected (i.e., evolution continues toward an optimum) but the path is not as direct when there are maternal effects, depending on the nature of the genetic covariance between the maternal and offspring trait. If it is positive, evolution will be enhanced. If it is negative, evolution will be slowed and may, temporarily, occur in a direction away from an optimum.

**Figure 4:**
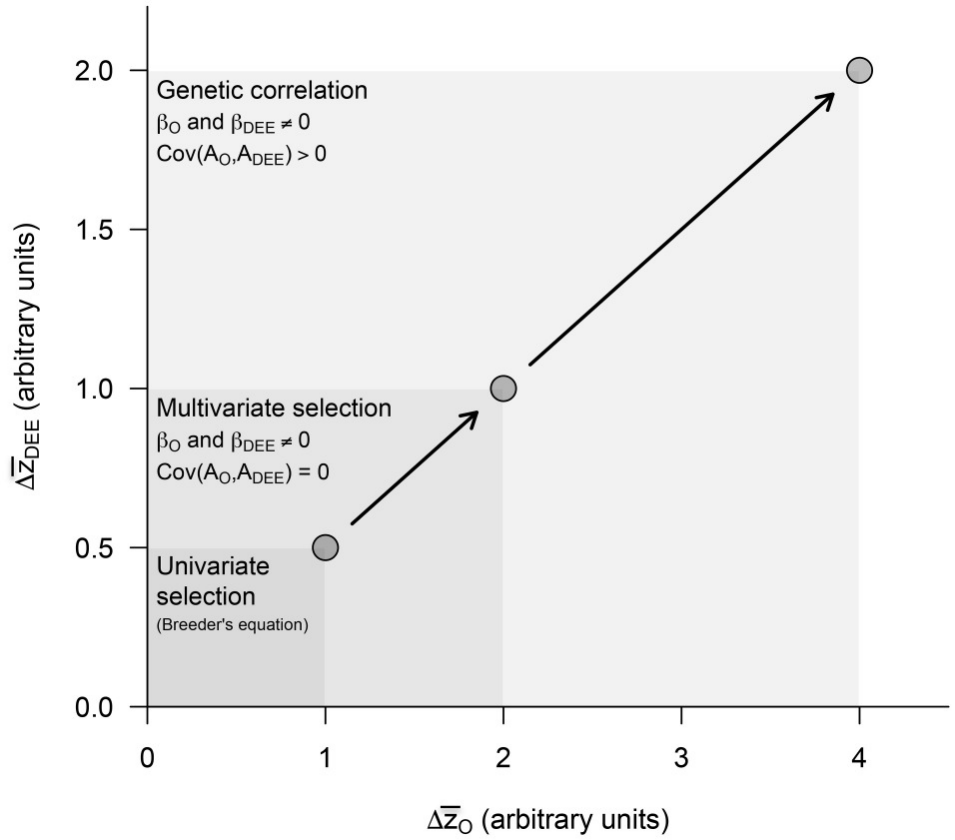
Predicted evolutionary responses in offspring growth rates ($\Delta {\bar{z}}_{O}$) and maternal DEE ($\Delta {\bar{z}}_{DEE}$) in increasingly complex evolutionary scenarios. The presence or absence of an effect was simulated by setting the different parameters to 0 or 1 (see Figures 2, 3). We start estimating evolutionary responses to univariate selection exerted separately in each of these traits (i.e., β_*DEE*_ = 0 in Equation 7a and β_*O*_ = 0 in Equation 7b) in the absence of genetic covariance [i.e., Cov(*A*_*O*_, *A*_*DEE*_) = 0], and then quantify the impact of multivariate selection and of a positive genetic covariance between traits. These two synergistic effects, which emerge from the standard quantitative genetic model including maternal effects, are expected to exacerbate evolutionary responses to selection in both traits and provide strong support to the parental care hypothesis for the evolution of endothermy.

Overall, these equations suggest that, depending on their sign and magnitude, selection gradients β_*O*_ and β_*DEE*_ and the genetic covariance Cov(*A*_*O*_, *A*_*DEE*_) between maternal and offspring traits can have synergistic effects (Figures 2, 3). During the evolution of endothermy, higher growth rates and DEE should be favored by selection, resulting in positive β_*O*_ and β_*DEE*_, and we would also expect Cov(*A*_*O*_, *A*_*DEE*_) to be positive because elevated assimilation rates should increase both growth rates and DEE. Under these circumstances, synergistic effects should exacerbate evolutionary responses of both parental and offspring phenotypes (Figure 4). This self-reinforcement process could be sustained until one of three things happen. First, there are no longer fitness benefits for faster growth rate (β_*O*_ = 0) or increased DEE levels (β_*DEE*_ = 0). Second, these traits reach a physiological limit (Bacigalupe and Bozinovic, 2002) so that *V*_*AO*_ and *V*_*A*(*DEE*)_ = 0. Third, the covariance Cov(*A*_*O*_, *A*_*DEE*_) between maternal and offspring phenotypes becomes negative due, for instance, to constraints in time and energy allocation (e.g., a trade-off between looking for food vs. taking care of the offspring).

## Discussion

In this article, we present a theoretical analysis for the evolution of endothermy by parental care by adapting a simple quantitative genetic model of maternal effects. The equations we present are discussed in detail in Cheverud and Moore (1994), see also Moore et al. (1998). Our model shows that maternal effects may have had an important contribution to the evolution of increased metabolic levels, due to a positive covariance between growth rates and DEE and synergistic effects of selection acting on these traits (Figures 2, 3). These would translate into greater evolutionary change per generation in both offspring and maternal traits (Figure 4) and are expected to result in elevated BMR—and eventually body temperature—as visceral organs increase in size and activity to maintain elevated assimilation rates (Koteja, 2000, 2004).

The evolutionary consequences of maternal effects have long been known in animal breeding (e.g., Falconer, 1965; Willham, 1972) and have been increasingly incorporated into an evolutionary framework (e.g., Cheverud, 1984; Kirkpatrick and Lande, 1989; Cheverud and Moore, 1994; Mousseau and Fox, 1998; Wilson and Réale, 2005; Räsänen and Kruuk, 2007). Unlike other abiotic environmental influences, the environmental influence exerted by mothers or close relatives is unique: if there is variation on the environment provided and if that variation results from genetic differences between individuals, the environment can have a heritable basis and evolve (Moore et al., 1997; Wolf et al., 1998). Three important evolutionary consequences arise from the environment having a genetic basis, as evidenced in our results. First, the rate and/or direction of phenotypic change in response to selection in the focal trait can be quite different from what would be predicted by standard quantitative genetics. Second, phenotypic evolution might not be constrained by the absence of heritability in the focal trait (Cheverud and Moore, 1994; Moore et al., 1997). And third, the synergistic effects of more than a single selective pressure on DEE and growth rates (Equations 7a,b) would not only drive faster rates of evolution of these traits, but might have also contributed to the continued directional sustained selection over longer periods of time required for the emergence of endothermy as we know it (Clarke and Pörtner, 2010).

Much of the mechanistic basis underlying the parental care hypothesis for the evolution of endothermy has been discussed previously, as well as the evidence available supporting this proposition (Farmer, 2000; Koteja, 2000). The main contribution of our model is to provide a formal assessment of the impact of parental care on the evolution of increased metabolic levels on the one hand and growth rates on the other. In this context, results indeed support the contention that parental care may have been a crucial factor behind the emergence of highly aerobic endothermic birds and mammals. According to our model, the presence of maternal effects may exacerbate the response to selection due to two independent effects that could act synergistically (Figure 4): a positive genetic covariance between maternal and offspring phenotypes, embedded in component Cov(*A*_*O*_, *A*_*DEE*_) (Equations 7a,b), and an environmentally-mediated component driving the correlated evolution of growth rates to selection on maternal DEE (β_*DEE*_ on Equation 7a) and vice-versa (β_*O*_ on Equation 7b). This result also leads to the counterintuitive possibility that growth rates and DEE could respond in tandem even when their genetic covariances were negative, as reported for maternal performance at weaning and offspring weight (Cheverud and Moore, 1994), although in this case evolutionary change may be seriously reduced (Kirkpatrick and Lande, 1989).

For tractability, our model treats DEE as a single trait. In reality, it is likely a composite trait. DEE is typically linked with multiple traits that influence parental care, such as BMR and assimilation rates (Koteja, 2000), as well as maternal body temperature and, indirectly, with incubation temperature (Farmer, 2000). While the association between DEE and BMR finds strong support in the literature (reviewed in Auer et al., 2017), how DEE is related to assimilation rates, body temperature and parental investment remains contentious (but see Sadowska et al., 2013, 2015). Confounding effects such as for instance, contrasting environmental temperatures, food availability, clutch size and the existence of multiple forms of parental care, preclude the establishment of general associations between these traits in extant lineages, let alone in transitional forms during the evolution of endothermy. Thus, while it might be argued that our model overestimates the importance of parental care, the main take-home message that synergistic evolutionary responses due to maternal effects may be substantially larger than predictions in the absence of these effects remains largely unchanged (see also Wolf and Wade, 2001).

Importantly, our model also highlights that selection on life-history traits that are mediated by maternal effects, such as growth rates and survival, can have important carryover effects in other aspects of the phenotype such as metabolic levels. While an increasing number of studies describe, for instance, the metabolic impact of changes in incubation temperatures in the offspring metabolic rates (e.g., Nord and Nilsson, 2011; DuRant et al., 2012; Sun et al., 2015), we contend that these effects may transcend early stages of ontogeny and have an impact on adult phenotypes and on evolutionary trajectories in the long term (Equations 7a,b). In this context, the role of maternal and developmental effects on metabolic and thermoregulatory performance during adulthood remains, we believe, virtually unexplored (but see Russel et al., 2008). In light of the comparative, mechanistic and theoretical evidence that support a key role of parental care during the evolution of endothermy, studying how maternal and developmental effects contributes to physiological variation during adulthood constitutes a promising venue for future research.

## Author contributions

Conception and design: LB and AM. All authors collaborated with the draft of the work and approved the final version of the manuscript.

### Conflict of interest statement

The authors declare that the research was conducted in the absence of any commercial or financial relationships that could be construed as a potential conflict of interest.
